# Glioblastoma adhesion in a quick-fit hybrid microdevice

**DOI:** 10.1007/s10544-019-0382-0

**Published:** 2019-03-21

**Authors:** Hsieh-Fu Tsai, Kazumi Toda-Peters, Amy Q. Shen

**Affiliations:** Micro/Bio/Nanofluidics Unit, Okinawa Institute of Science and Technology Graduate University, 1919-1 Tancha, Onna-son, Okinawa 9040495 Japan

**Keywords:** Glioblastoma adhesion, Endothelium, Bubble-free, Electric field, Shear flow, Multiplexing

## Abstract

Translational research requires reliable biomedical microdevices (BMMD) to mimic physiological conditions and answer biological questions. In this work, we introduce a reversibly sealed quick-fit hybrid BMMD that is operator-friendly and bubble-free, requires low reagent and cell consumption, enables robust and high throughput performance for biomedical experiments. Specifically, we fabricate a quick-fit poly(methyl methacrylate) and poly(dimethyl siloxane) (PMMA/PDMS) prototype to illustrate its utilities by probing the adhesion of glioblastoma cells (T98G and U251MG) to primary endothelial cells. In static condition, we confirm that angiopoietin-Tie2 signaling increases the adhesion of glioblastoma cells to endothelial cells. Next, to mimic the physiological hemodynamic flow and investigate the effect of physiological electric field, the endothelial cells are pre-conditioned with concurrent shear flow (with fixed 1 Pa shear stress) and direct current electric field (dcEF) in the quick-fit PMMA/PDMS BMMD. With shear flow alone, endothelial cells exhibit classical parallel alignment; while under a concurrent dcEF, the cells align perpendicularly to the electric current when the dcEF is greater than 154 V m^− 1^. Moreover, with fixed shear stress of 1 Pa, T98G glioblastoma cells demonstrate increased adhesion to endothelial cells conditioned in dcEF of 154 V m^− 1^, while U251MG glioblastoma cells show no difference. The quick-fit hybrid BMMD provides a simple and flexible platform to create multiplex systems, making it possible to investigate complicated biological conditions for translational research.

**Electronic supplementary material:**

The online version of this article (10.1007/s10544-019-0382-0) contains supplementary material, which is available to authorized users.

## Introduction

In biomedical microdevices (BMMDs), biochemical and biophysical microenvironments can be manipulated by combining device design and operation conditions to probe biochemical and biophysical properties or to investigate biological phenomena. Practical BMMD designs must consider system integration and fabrication process to reduce the production volume and cost, ensure the sterilization and user-friendly operation (McRae et al. 2016). Common materials used in fabrication of BMMDs include different types of thermoplastics, transparent and gas permeable silicone rubbers, glasses, and perfluoropolymers (Ren et al. 2013). Transparent rigid thermoplastics, such as poly(methyl methacrylate) (PMMA) and cyclic olefin copolymers (COC), and transparent elastomeric silicone rubber poly(dimethyl siloxane) (PDMS) are particularly favored in BMMD production in laboratory settings (Cheng et al. 2004; Prokop et al. 2004; Steigert et al. 2007). Each material has its distinct advantages and disadvantages (see summary in Table 1). By using thermoplastics, fabrication cost is low while bonding strategies and interconnect choices are more readily available for constructing the world-to-chip interface. Fabrication of complex three dimensional (3D) internal structures is also possible, but the spatial resolution of direct-writing methods is inferior in comparison to lithographic methods (Cheng et al. 2004). Alternatively, micrometer-precision with PDMS substrates is possible through the soft lithography process, although the process is usually limited to quasi-planar (2.5D) microstructures (Xia and Whitesides 1998). PDMS is also flexible and highly gas permeable, making it suitable to fabricate BMMDs with active components such as microvalves, and to create gas concentration gradients for automated lab-on-chip applications. Although fabrication of complex 3D microstructures in PDMS BMMDs is possible, it requires complicated multi-step fabrication, alignment, and off-stoichiometry bonding (Thorsen et al. 2002). Moreover, PDMS has very low surface energy, which limits its bonding and world-to-chip interconnect choices.

**Table 1:** Common materials to fabricate BMMDs

	Thermoplastics (PMMA, COC)	PDMS	Glass (Industrial)
Advantages	1. 3D fluidics by stacking	1. High spatial resolution	1. High spatial resolution
	2. Low gas permeability	by lithography (1 *μ*m)	by etching (10 nm)
	3. Versatile world-to-chip	2. High gas permeability	2. Excellent chemical stability
	connection	3. Active components possible	3. High transparency
		4. Easy priming to remove bubbles	4. High rigidity to sustain
			high flow rates
Disadvantages	1. Low spatial resolution	1. Limited to 2.5D structures	1. Limited to 2.5D structures
	by laser cutting (100 *μ*m)	2. Adsorption/absorption of	2. Facility requirement for
	2. High *μ*m-scale roughness	hydrophobic chemical species	wet or dry etching
	by micromilling	3. Limited bonding strategies	3. Special bonding requirement
	3. Birefringent	and substrate choice due	4. Brittle
	4. Rigid structures only	to low surface energy	
	5. Prone to trap air bubbles	4. Limited world-to-chip	
	6. Medium solvent	interface choice	
	compatibility	5. Poor solvent compatibility	

For successful and reliable cell culture in BMMDs, it is essential to ensure sterility, controlled microenvironment, and guided reagent delivery with bubble-free conditions (Kim et al. 2007). Bubble prevention is crucial because microbubbles can impose strong interfacial tensions that shear adherent cells or cause cell damage in the microchannel. In thermoplastic BMMDs, to minimize air bubbles, the device is often pre-assembled with reagent delivery components and a world-to-chip interface prior to seeding cells into the device. As a result, the dead volume tends to be large in pre-assembled thermoplastic BMMDs, which leads to longer reagent delivery time to cells (Tsai et al. 2012). In PDMS BMMDs, although a delicate buffer exchange procedure can be adapted to prevent air bubbles in microchannels (Wang et al. 2012), the hydrophobic nature of PDMS often causes air bubble accumulation at world-to-chip interconnects, which can easily propagate into microchannels. Moreover, the elastic nature of PDMS is prone to leakage at interconnects (Christensen et al. 2005).

In this work, we present a hybrid quick-fit PMMA/PDMS BMMD, combining the advantages and mitigating the disadvantages of PMMA and PDMS to create an air-tight but reversibly sealed cell culture platform with low reagent dead volumes. Reversibly sealed BMMDs are empowered by mechanical or vacuum sealing of an elastomeric PDMS chip to a rigid substrate, thus allowing the BMMD to be sealed and dismantled quickly before and after each experiment (Khademhosseini et al. 2005; Bang et al. 2006; Skafte-Pedersen et al. 2012; Uzel et al. 2016; Abhyankar et al. 2016). This hybrid device enables further cellular treatment (i.e., fixation and immuno-staining) in the BMMD. To validate this setup, we choose glioblastoma and endothelial cells as a cell-cell interaction model system. Glioblastoma, the most common primary high-grade brain tumor type in adults, can diffuse and metastasize intracranially through white matter tracts or defined perivascular structures, such as blood vessels and the subarachnoid space (Holland 2000; Segarra et al. 2015). Although intravasation and extracranial metastasis in glioblastoma seldom occur (Bernstein and Woodard 1995; Beauchesne 2011), endothelial cells and the associated blood-brain barrier contribute greatly to establish and maintain the tumor microenvironment of glioblastoma (Cuddapah et al. 2014; Watkins et al. 2014). An *in vitro* cell-cell interaction model of glioblastoma and endothelial cells could further our understanding of the perivascular tumor microenvironment of glioblastoma (Tsai et al. 2017). Endothelial cells cultured *in vitro* can be conditioned chemically or physically through mechanical or electrical stimulation to up-regulate expression of cell adhesion molecules or to promote cell morphology with more natural physiological conditions (Sheikh et al. 2003; Zhao et al. 2004; Bai et al. 2011; Khan and Sefton 2011; Uzarski et al. 2013; Jaczewska et al. 2014; Davis et al. 2015). We employ the quick-fit BMMD to demonstrate its robustness by applying concurrent electrical and mechanical conditioning on the endothelial cells and further investigate the adherence of glioblastoma cells on the conditioned endothelium. The design and fabrication of a shear flow and electric field co-stimulation microfluidic chip with the quick-fit hybrid BMMD is discussed in Sections 2.1–2.2. The adhesion of glioblastoma to endothelial cells in a static condition and in a coexisting shear flow and electric field microenvironment are discussed in Sections 2.3–2.7 and Section 3. Conclusion is provided in Section 4.

## Materials and methods

### Concurrent shear flow and electric field chip design

**Fig. 1:**
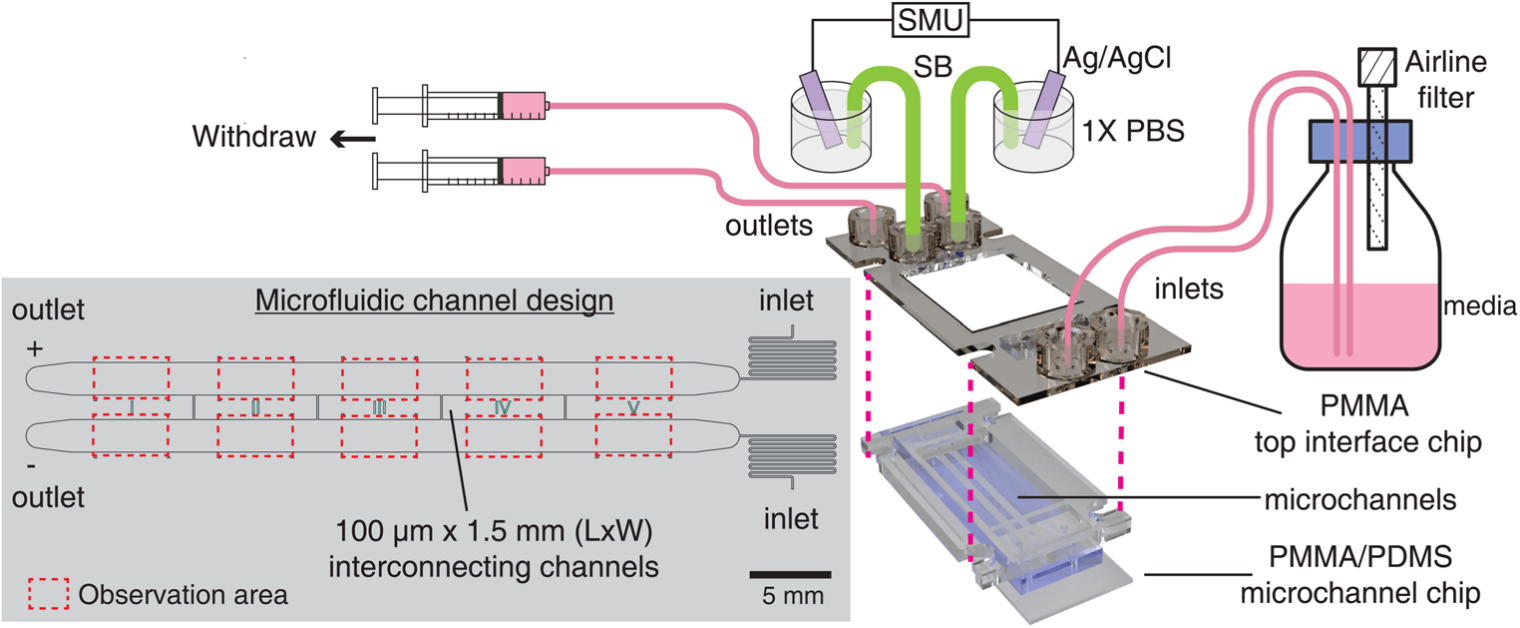
Experimental setup of concurrent shear flow and electric field conditioning of endothelial cells in a *shear flow and electric field co-stimulation microfluidic chip* (SFEFC). Endothelial cells are cultured in the bottom PMMA/PDMS microchannel device in a user-friendly manner. To pre-condition the cells, the PMMA/PDMS chip is reversibly sealed with the top PMMA interface chip before applying the shear flow and electric field. After conditioning, the chip can be easily recovered. SMU: source measure unit; SB: salt bridge. Detailed configuration of PMMA top interface chip and PMMA/PDMS microchannel chip can be found in Fig. 3

The shear flow and electric field co-stimulation microfluidic chip (SFEFC) was designed to quick-fit a top PMMA interface chip with a bottom PMMA/PDMS microchannel device where cells were cultured (see schematic in Fig. 1). The SFEFC was constructed to create multiple electric fields in an R-2R resistor ladder configuration (Tsai et al. 2012; Zhao et al. 2014). Two 2 mm-wide main microchannels with interconnected 100 *μ*m by 1.5 mm (L×W) channels at a spatial interval of 7.5 mm in SFEFC created 10 channel segments with various electric field strengths (EFSs) (Fig. 1). The segments on the top side of SFEFC had electric current vector flowing against the shear flow direction, while the segments on the bottom side of SFEFC had electric current vector flowing along the shear flow direction. This multiplex configuration provided a platform for high-throughput screening of cellular responses to coexisting shear flow and electric field. Cells were observed only in the 4.5 mm-long observation area, in the middle of each segment, 1.5 mm from the interconnection channels (where the electric fields were stable).

**Fig. 2:**
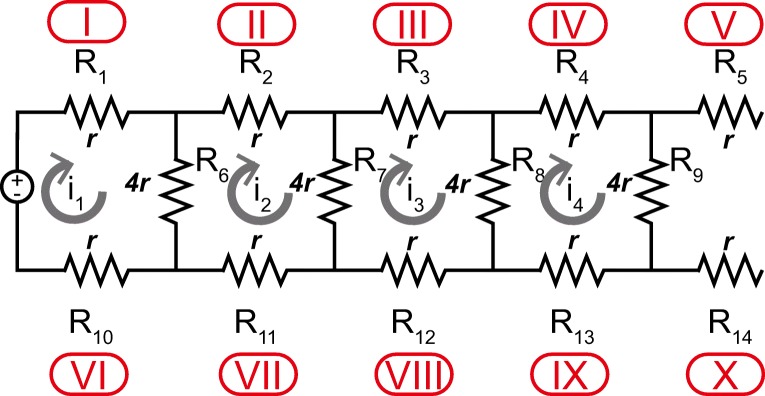
The equivalent circuit of electric field in the *shear flow and electric field co-stimulation microfluidic chip* (SFEFC). Each microfluidic channel segment can be regarded as a flow resistor and an electrical resistor with relative electrical resistances can be calculated according to the Ohm’s law

The electrical equivalent circuit of SFEFC is shown in Fig. 2. Each segment of the microfluidic channel network was regarded as an electrical resistor in which relative electrical resistances were calculated and modeled by Ohm’s law and Kirchhoff’s circuit laws. In the equivalent circuit, the endpoint of R_5_ and R_14_, the adjacent segments from both inlets, were open in the electric circuit. No electric current was flowing through them. Cells in the two segments were only subjected to shear flow.

According to Ohm’s law, the electrical resistance of a resistor, *R*, is proportional to the length and inversely proportional to the cross-sectional area:

1 $$ R=\rho\frac{L}{A}=\frac{\rho L}{W H},  $$

where *ρ*, *L*, *A*, *W*, and *H* are the electrical resistivity of the medium, the length, the cross-sectional area, the width, and the height of the microchannel, respectively. Assuming the electrical resistance of R_1_ being *r*, the relative electrical resistances of other segments (R_2_ : R_14_) can be calculated accordingly.

The electric current flowing through each resistor was calculated by Kirchhoff’s circuit law and simulated in the electronic design automation software (OrCAD Lite, Cadence Design Systems, USA) by:

2a $$ \begin{array}{@{}rcl@{}} 10r\times \text{i}_{2}-4r\times \text{i}_{1}-4r\times \text{i}_{3}&=&0, \end{array} $$

2b $$ \begin{array}{@{}rcl@{}} 10r\times \text{i}_{3}-4r\times \text{i}_{2}-4r\times \text{i}_{4}&=&0, \end{array} $$

2c $$ \begin{array}{@{}rcl@{}} 10r\times \text{i}_{4}-4r\times \text{i}_{3}&=&0. \end{array} $$

By solving the system of equations in Eq. 2c, the ratio of electric currents between each segment, hence the ratio of electric field strengths, was derived in Eq. 3c:

3a $$ \begin{array}{@{}rcl@{}} \text{i}_{1}:\text{i}_{2}:\text{i}_{3}:\text{i}_{4} &=& \text{E}_{\text{I}}:\text{E}_{\text{II}}:\text{E}_{\text{III}}:\text{E}_{\text{IV}}:\text{E}_{\text{V}} \end{array} $$

3b $$ \begin{array}{@{}rcl@{}} &=&\text{E}_{\text{VI}}:\text{E}_{\text{VII}}:\text{E}_{\text{VIII}}:\text{E}_{\text{IX}}:\text{E}_{\text{X}} \end{array} $$

3c $$ \begin{array}{@{}rcl@{}} &=&10.5:5.2:2.5:1:0 \end{array} $$

To calculate the flow rate and electric current applied to condition endothelial cells, the dynamic shear viscosity and the electrical conductivity of the endothelial cell growth medium (ECGM, C22110, PromoCell GmbH, Germany) were characterized experimentally. The dynamic shear viscosity was measured by using an A05 sensor in a microfluidic viscometer (m-VROC, Rheosense, USA). The ECGM was verified as a Newtonian fluid within the flow rate regimes of interest (shear rate ranging 293–1334 s^− 1^). The dynamic shear viscosity was measured as 0.757 ± 0.012 mPa.s (mean ± standard deviation) under a constant shear stress of 1 Pa and 37 ^∘^C. The electrical conductivity of ECGM was characterized by using a water quality meter with a calibrated conductivity cell (3552-10D on LAQUA F-74, Horiba, Japan). The electrical conductivity of ECGM at 37 ^∘^C was measured as 1.444 ± 0.001 S m^− 1^ (mean ± standard deviation).

The Stokes flow and electric field in SFEFC were simulated by solving steady-state Navier-Stokes equations and Maxwell’s equations in COMSOL Multiphysics software (v5.2, COMSOL, USA). An aqueous medium with dynamic shear viscosity of 0.75 mPa.s, electrical conductivity of 1.44 S m^− 1^, density of 1000 Kg m^− 3^, and a dielectric constant of 80 were used for the ECGM (Tsai et al. 2016).

The simulated EFS ratio, when an electric current of 86.4 *μ*A was carried from one outlet of the SFEFC to the other outlet, is shown in Eq. 4b:

4a $$ \begin{array}{@{}rcl@{}} \text{E}_{\text{I}}:\text{E}_{\text{II}}:\text{E}_{\text{III}}:\text{E}_{\text{IV}}:\text{E}_{\text{V}} & =&\text{E}_{\text{VI}}:\text{E}_{\text{VII}}:\text{E}_{\text{VIII}}:\text{E}_{\text{IX}}:\text{E}_{\text{X}} \end{array} $$

4b $$ \begin{array}{@{}rcl@{}} & =& 9.72:4.97:2.44:1:0 \end{array} $$

### Device fabrication

**Fig. 3:**
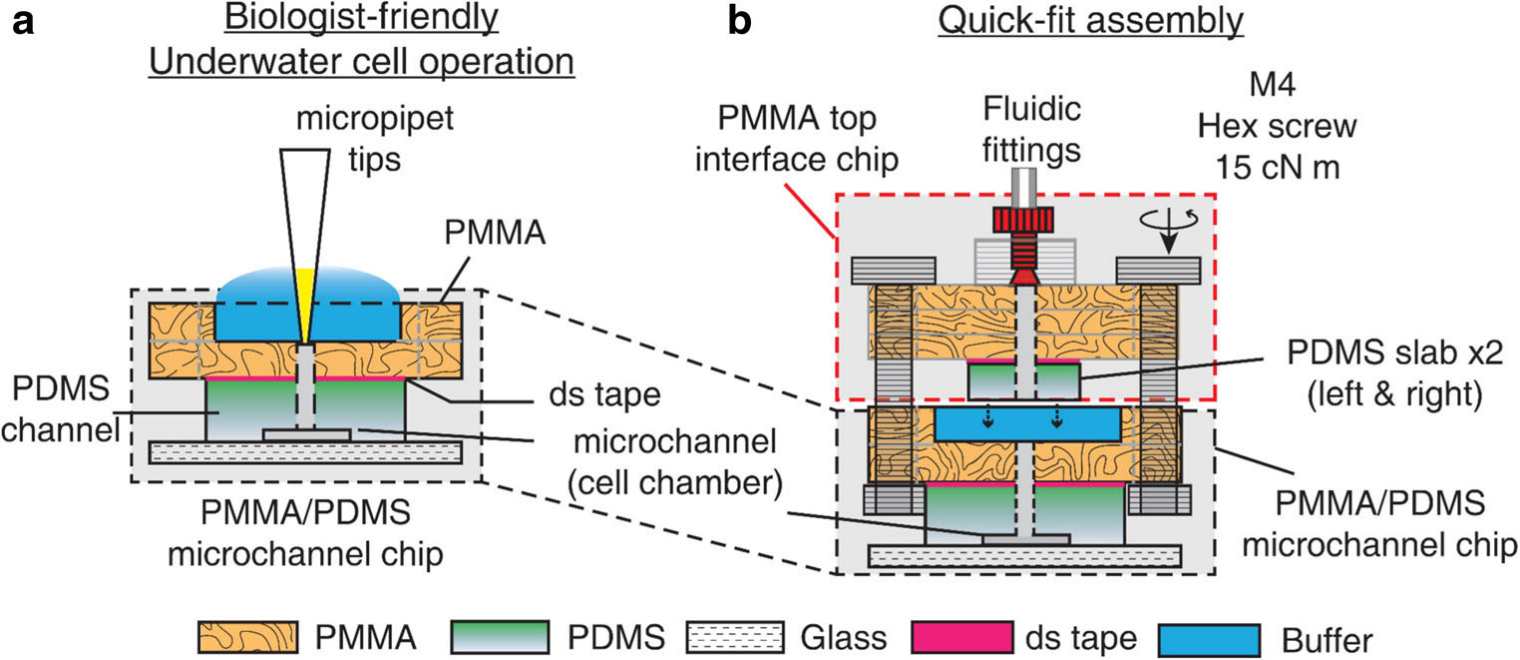
Liquid manipulation on the SFEFC. **a** Operator-friendly underwater fluid manipulation at early stage of chip preparation using the PMMA/PDMS microchannel chip. Cells are cultured in the microchannels in the PMMA/PDMS chip; **b** The complete chip can be assembled just prior to on-chip experiments. The PMMA top interface chip is shown in red dashed box. PDMS slabs on the PMMA top interface chip compress and seal the interface to the PMMA/PDMS microchannel chip (inside the black dashed box). After each experiment, the cells in the PMMA/PDMS microchannel chip can be easily recovered after disassembly

The PMMA interface chip (Figs. 1 and 3) was fabricated by patterning fluidic connections, inlets/outlets, salt bridge (SB) connections, and M4 screw clamp slots on three 0.5 mm thick PMMA sheets (CM-205, Chi-Mei Corp, Taiwan), by using a CO_2_ laser scriber (VLS3.60, Universal Laser Systems, USA). The PMMA sheets were joined by thermal bonding, as previously described by Tsai et al. (2012, 2016). Adapters with M6 threads (SPC-M6-C, Nabeya, Japan) were glued onto the PMMA chip using a UV adhesive (3301, Loctite, USA). PDMS slabs, 2 mm thick, punched with inlet and outlet holes were affixed to the interface chip using a dual-energy silicone/acrylic double-sided tape (85 *μ*m thick, No. 5302A, Nitto Denko, Japan) (Carlborg et al. 2010).

Similar to the interface chip, the PMMA component of the PMMA/PDMS microchannel chip (Figs. 1 and 3) was fabricated by patterning liquid reservoirs, inlets, outlets, and screw clamp slots on two 2 mm-thick PMMA sheets. The PMMA pieces were again thermally bonded. The PDMS channel chip was fabricated by the soft lithography method (Xia and Whitesides 1998). Briefly, a chrome photomask was patterned with microchannels through a maskless writer (DL-1000, Nanosystem Solutions, Japan), developed, and etched. A 100 *μ*m-thick SU-8 3025 template of microchannels was patterned on a silicon wafer using a mask aligner (MA/BA6, SUSS MicroTec, Germany) with the chrome photomask. After the template was treated with perfluorosilane, PDMS resin (Sylgard 184, Dow Corning, USA) was sandwiched between the SU-8 3025 template and a 15 mm-thick PMMA sheet with a 4 mm-thick PMMA spacer in between to ensure parallelism and flatness of both surfaces. The PDMS resin was cured at 60 ^∘^C for 4 h to fabricate the PDMS component. The PMMA/PDMS chip was assembled by joining the bonded PMMA component and the PDMS microchannel component with the silicone/acrylic double-sided tape. The PMMA/PDMS microchannel chip was completed by bonding the joined PMMA/PDMS piece to a glass substrate (60 × 24 mm, No. 1.5H, Paul Marienfeld GmbH, Germany) after O_2_ plasma treatment (AP-300, Nordson MARCH, USA). The glass substrate was washed thoroughly in an industrial cleaning solution (1% TFD4, Franklab, France), deionized water, and dried under nitrogen gas prior to bonding.

### Cell culture and maintenance

Primary human umbilical vein endothelial cells (HUVECs) were cultured in the endothelial cell growth medium (C12203, PromoCell GmbH, Germany). T98G (CRL-1690, ATCC, USA) and U251MG (IFO50288, JCRB, Japan) glioblastoma cells were cultured in minimum essential medium *α* (MEM*α*, 12000022, Gibco, USA) supplemented with 2.2 gL^− 1^ NaHCO_3_ and 10% fetal bovine serum (FBS, Invitrogen, USA). All cells were cultured in a humidified 5% CO_2_ atmosphere (Forma Steri-cycle i160, Thermofisher, USA) and passaged whenever confluency reached 80%. Cells from passages 2 to 8 were used in the adhesion study. Red fluorescent protein-expressing clones of T98G (T98G-dsRed) and U251MG (U251MG-dsRed) cells were established by transfecting a plasmid which expressed translation elongation factor-1 alpha conjugated with a red fluorescent protein into the two cell lines (EF1alpha-DsRed-Express2, 631979, Takara Bio, Japan). Stable fluorescent clones were selected by subculture in media containing 500 *μ*g mL^− 1^ G418 and aseptically sorted by flow cytometry (Aria II, BD, USA).

### Glioblastoma adhesion on statically cultured endothelium

In a static adhesion experiment, glass bottoms of 8-well chambered slides (Lab-tek II, Nunc, USA) were coated with 10 *μ*g mL^− 1^ of human plasma fibronectin (Invitrogen, USA). HUVEC cells of 1 × 10^5^ were seeded in each well and allowed to adhere for 24 h. Angiopoietin 1 (Ang1), an endothelial cell-specific tyrosine kinase receptor ligand that upregulates adhesion molecules on glioblastoma cells was purchased from Peprotech USA. Both glioblastoma cells were incubated with 100 ng mL^− 1^ of Ang1 for 24 h prior to adhesion experiments. Activated endothelial cells were prepared by incubating HUVECs with 10 ng mL^− 1^ tumor necrosis factor *α* (TNF*α*) for 24 h (Invitrogen, USA). Prior to adhesion experiments, confluent HUVECs were labeled by incubation with 1 *μ*M CellTracker Green CMFDA dye (C2925, Invitrogen, USA) for 15 min and washed once with Dulbecco’s phosphate buffered saline (D-PBS).

To commence static adhesion experiments, T98G-dsRed and U251MG-dsRed cells were trypsinized and resuspended in ECGM. The two glioblastoma cell lines were seeded at glioblastoma (2) : HUVEC (1) ratio. Glioblastoma cells were allowed to adhere to endothelial cells for 1 h at 37 ^∘^C. Unadhered cells were washed away by gentle pipetting with D-PBS. Cells were fixed with 4% paraformaldehyde in D-PBS immediately and imaged using an epifluorescence microscope with filter sets for FITC and TRITC channels. Densities of adhered glioblastoma cells were counted and averaged over four 1 mm^2^ fields in the middle of each well (16 fields total). All experiments were performed in triplicates and the data were represented as the mean ± 95% confidence interval, which is 1.96 standard errors of the mean. One-way analysis of variance with Tukey’s post-hoc multiple-comparison tests were performed on collected data using Prism 6 (Graphpad, USA). The confidence level to reject a null hypothesis between two data sets was set at 95%. A p-value (P), the probability for a true null hypothesis less than 0.05 represents a statistical significance at 95% confidence.

### SFEFC assembly and endothelium conditioning by external fields

The usage of quick-fit hybrid devices consists of two steps: user-friendly on-chip cell culture and quick-fit assembly. Briefly, in the first step, users can prepare cells and culture them in the microchannels on the PMMA/PDMS microchannel chip (Fig. 3a). Bubble-free cell culture is achieved by an underwater cell operation workflow. In the second step, prior to initiation of shear flow conditioning and electric field stimulation, the two PDMS slabs on the PMMA top interface chip are aligned to the inlets and outlets of the PMMA/PDMS microchannel chip. Through the compressive force applied by tightening hex screws, the PDMS slabs deform slightly to seal the interface to the PMMA piece of the PMMA/PDMS microchannel chip (Fig. 3b). Moreover, the reagents are primed in the channels of the PMMA interface chip, thus, after quick-fit assembly, the dead volume is reduced and the reagents can be delivered to cells with reduced delivery time.

To prepare confluent HUVEC culture, the PMMA/PDMS chip was filled with 99.5% ethanol (Wako, Japan) to remove bubbles, as described by Wang et al. (2012). The solution in the microchannels was next replaced by deionized water and D-PBS. The buffer held in fluid reservoirs on the PMMA part of the PMMA/PDMS chip (Fig. 3a) ensured that no bubbles were trapped at the interface of the inlets and outlets and prevented accidental bubble injection into the microchannels that would cause disruption of microfluidic flow and cell death.

At an early stage of preparing cells, no complex tubing connections were required. Manipulation of the PMMA/PDMS chip was user-friendly because micropipet tips could be used to deliver fluids and cells by either active pressure delivery or by gravity driven flow (see Video S1 in supplementary information). Furthermore, the fluid volume needed to fill the microchannels was very low (on the order of microliters), so the amount of extracellular matrix protein and the number of cells required were limited.

To start endothelium culture in the PMMA/PDMS chip, the glass bottom was coated with 10 *μ*g mL^− 1^ of human plasma fibronectin for 2 h. HUVEC cells at concentration of 10^7^ cells mL^− 1^ were injected into the microchannels and allowed to adhere for 3 h.

To assemble the complete quick-fit microfluidic chip, the fluidic tubing and salt bridges (1.5% agarose in D-PBS) were first connected to the PMMA interface chip and primed with cell culture media. The PMMA interface chip was then brought into contact with PMMA/PDMS microchannel chip and sealed tight with four M4 stainless hex screws and nuts. The four screw clamp slots are located on both the PMMA interface chip and PMMA/PDMS microchannel chip so that even pressure can be applied to the PDMS slabs on the PMMA interface chip. The SFEFC was assembled after a tight seal was achieved by applying 15 cN m of torque on the four hex screws with a torque driver (RTD60CN, Tonichi, Japan) (Fig. 3b). The pressure applied on the PDMS slabs deforms the slabs and seals the interface between the top interface chip and the microchannel chip. After each experiment, the cells in the PMMA/PDMS chip could be quickly recovered by removing the hex screws. In addition, the PMMA interface chip could be reused to assemble with another PMMA/PDMS chip to increase experimental throughput with low liquid dead volume.

#### Endothelium conditioned by shear flow

The assembled SFEFC with a confluent HUVEC cell layer in the microchannel was set up in the incubator as shown in Fig. 1 and supplementary Fig. S.1. We conditioned the endothelium by shear flow mimicking the physiological conditions in hemodynamic flows (Malek et al. 1999). The shear stress imposed on the endothelial cells in a rectangular channel can be calculated according to the modified Hagen-Poiseuille equation Eq. 5 (Gaver and Kute 1998):

5 $$ (\tau^{*}_{s})_{max} = 2.95\times \frac{6\mu\text{Q}}{W\times H^{2}},  $$

where *μ*, *Q*, *W*, and *H* are the dynamic shear viscosity, volumetric flow rate, width, and height of the rectangular microchannel, respectively. Primary endothelial cells like HUVECs are reported to respond to shear flow and align parallel to the flow direction when shear stress exceeds 10 dynes cm^− 2^ (equivalent to 1 Pa) (Buchanan et al. 2014; Abaci et al. 2014). Based on Eq. 5, to impose 1 Pa shear stress on the endothelial cells in a 2 mm-wide and 100 *μ*m-high microchannel filled with a cell culture medium with dynamic viscosity of 7.57 × 10^− 4^ Pa s, the flow rate should be 1.49 *μ*L s^− 1^ (equivalent to 5.4 mL h^− 1^).

Specifically, the flow was delivered by pulling ECGM supplemented with antibiotics cocktail (1X PSN, Invitrogen, USA) from a 250 mL serum bottle by two 50 mL syringes (Terumo, Japan) using a dual channel syringe pump (YSP-202, YMC, Japan). The syringe pump was controlled remotely with the control software (Syringepump Pro, USA). The flow rate was set starting at 0.1 mL h^− 1^ and doubled every 3 h until the flow rate reached 5.4 mL h^− 1^.

#### Endothelium conditioned by both shear flow and electric field

Once the flow rate reached 5.4 mL h^− 1^ (corresponding to the shear stress of 1 Pa), a direct current electric field (dcEF) was applied to HUVECs in sections I to V and VI to X at 300:153.6:75.5:30.9:0 respectively based on the simulation results (described in Section 2.1). Specifically, the dcEF was applied by a source measure unit (2410, Keithley, USA) through two silver/silver chloride electrodes in D-PBS where the salt bridges were immersed (Fig. 1). The simultaneous conditioning of the HUVECs with shear flow and electric field were carried out for 24 h prior to glioblastoma cell adhesion experiments. Upon filling of the syringes, the content was discarded prior to restarting the syringe pump.

### Glioblastoma adhesion experiments on conditioned endothelium

After shear flow and dcEF conditioning, the PMMA interface chip was immediately removed by unscrewing M4 Hex screws. The confluent HUVECs were labeled by incubation with 1 *μ*M CellTracker Green for 15 min and washed once with 1X D-PBS before the glioblastoma cell adhesion experiment. T98G-dsRed and U251MG-dsRed cells were trypsinized and suspended in ECGM. The two types of glioblastoma cells were seeded at 2× 10^6^ cells mL^− 1^ in 200 *μ*L by gravity-driven flow. Glioblastoma cells were allowed to adhere for 1 h at 37 ^∘^C. Unadhered cells were washed away with 200 *μ*L of D-PBS by gravity flow. Cells were fixed with 4% paraformaldehyde in D-PBS and imaged with an epifluorescence microscope. Cell counting and statistical inferences in these adhesion experiments folllowed the same statistical measurements as those on statically cultured endothelium (more details in Section 2.4).

### Immunofluorescence staining

The presence of glial fibrillary acidic protein (GFAP) and Tie2 receptor in T98G-dsRed and U251MG-dsRed cells were examined by immunofluorescence staining. Both cells were fixed with 4% paraformaldehyde and permeabilized with 0.1% Triton X-100 in D-PBS. Fluorophore-conjugated species-specific secondary antibodies against the primary antibodies were used to detect the two proteins.

The expression of CD31 membrane adhesion molecule (PECAM-1) and cytoskeleton F-actin of conditioned endothelial cells was also characterized by immunofluorescence staining on chip following the same fixation and permeabilization protocol described above. The detection of CD31 and F-actin was conducted with an anti-CD31 primary antibody and a fluorophore-conjugated secondary antibody (NBP1-71663SS, Novus Biological, USA & A21467, Invitrogen, USA) as well as the fluorophore-conjugated phalloidin (A12380, Invitrogen, USA). The stained cells were scanned under a confocal laser scanning microscope with a 10X objective (A1R+, Nikon, Japan).

## Results and discussion

### Glioblastoma adhesion to endothelium in static condition

**Fig. 4:**
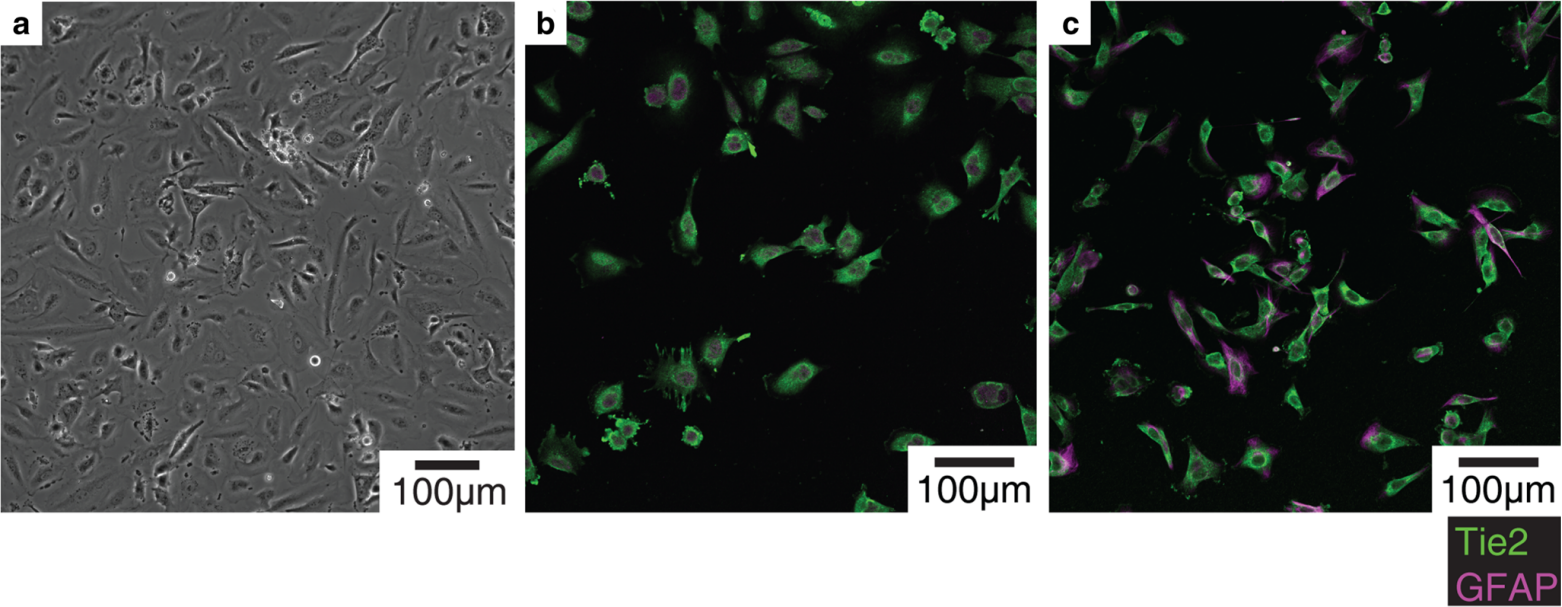
Microimages of endothelial cells and glioblastoma cells: **a** Endothelial cells cultured in static condition show random orientation. Both **b** T98G and **c** U251MG glioblastoma cells exhibit both GFAP and Tie2 expressions

*Phenotyping endothelial cells and glioblastoma cells cultured in static condition:* Prior to adhesion experiment, the phenotypes of endothelial cells and glioblastoma cells in static condition were verified. Endothelial cells cultured in static condition expressed random orientation (Fig. 4a). The immunofluorescence staining confirmed that both T98G and U251MG glioblastoma cells exhibited both GFAP and Tie2 expressions, indicating a glial phenotype with potential angiopoietin/Tie2 signaling (Fig. 4b and c).

**Fig. 5:**
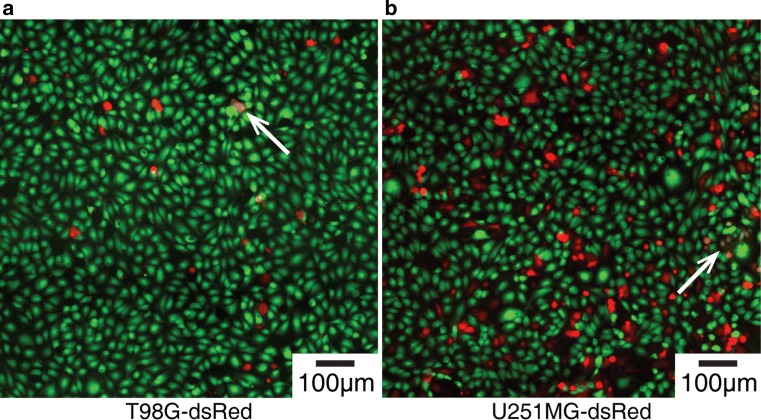
The adhesion of **a** T98G-dsRed, and **b** U251MG-dsRed, to confluent endothelium (fluorescently labeled green). The cells with yellow color (next to the white arrows) indicate the colocalization of CellTracker Green dye in endothelial cells and dsRed fluorescent protein in glioblastoma cells. The colocalization implies cell fusion or intercellular exchange events that may be important in the perivascular microenvironment of glioblastoma

**Fig. 6:**
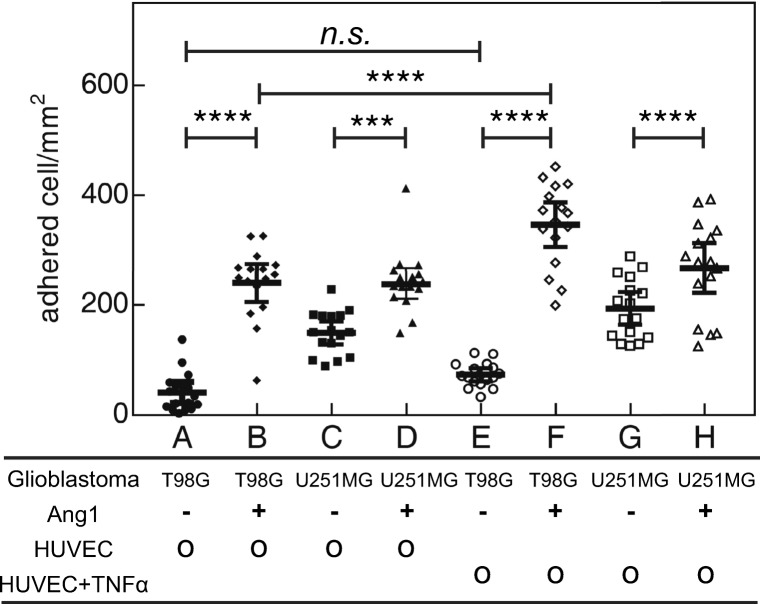
The adhesion of T98G-dsRed and U251MG-dsRed to endothelium under the stimulation with Ang1 and TNF*α* in static condition. *** denotes P < 0.001; **** denotes P < 0.0001; n.s. denotes no significance

*Glioblastoma cell adhesion to endothelial cells in static condition:* Liu et al. (2010) reported that U87MG and U251MG cells adhered to endothelial cells through Ang1/Tie2 signaling. We first tested the adhesion of T98G and U251MG glioblastoma cells under similar conditions. The adhesion of T98G-dsRed and U251MG-dsRed glioblastoma cells to confluent endothelial cells was examined (Fig. 5). After 1 h of adhesion, glioblastoma cells adhered to the endothelial and displaced them. The adhesion of glioblastoma cells with and without Ang1 to confluent HUVECs with and without activation with TNF*α* were quantified and statistically examined in Fig. 6 (microscopy images are shown in supplementary Figs. S.2 & S.3).

Without Ang1 treatment, adhesion of U251MG-dsRed to endothelium was greater than that of T98G-dsRed (P < 0.0001). Ang1 treatment increased the adhesion of both T98G-dsRed and U251MG-dsRed to endothelium (P < 0.001). This is consistent with existing reports that glioblastoma cells demonstrate enhanced interaction with endothelial cells via Ang1/Tie2 signaling (Liu et al. 2010). TNF*α* is a cytokine known to upregulate adhesion molecules on endothelial cells (Mackay et al. 1993; Jaczewska et al. 2014). However, only T98G cells activated with Ang1 showed increased adhesion to TNF*α*-activated HUVECs but not U251MG cells. This result suggests that the Ang1/Tie2 signaling of glioblastoma may not directly cross-talk with TNF*α* signaling-related adhesion molecules expressed on HUVECs. Further identification of the adhesion molecules is needed.

**Fig. 7:**
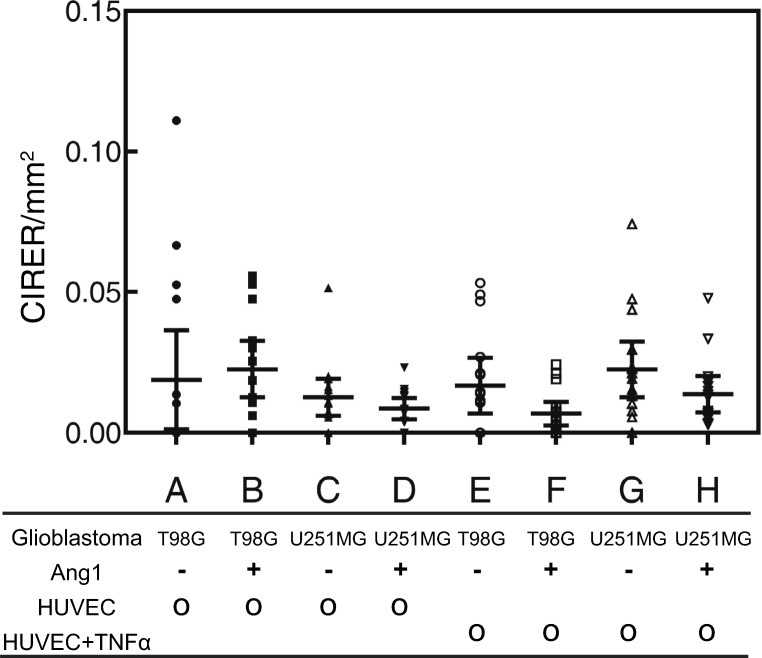
The calibrated intercellular transport event ratio (CITER) observed in static condition calibrated by adhesion count

In addition to glioblastoma adhesion, colocalization events were observed between the dsRed fluorescence of glioblastoma and CellTracker Green fluorescence of endothelial cells *in vitro* (Fig. 5). Colocalization events were quantified and calibrated by adhesion events as shown in Fig. 7 and supplementary Fig. S4. The colocalization events did not increase or decrease (P > 0.05) due to Ang1 stimulation or TNF*α* activation, although Ang1-stimulation increased the adhesion in both glioblastoma cell lines. Interestingly, more colocalization events were detected in Ang1-stimulated T98G-dsRed but not in U251MG-dsRed (see Fig. S.4, P < 0.0001).

Colocalization events suggest that glioblastoma cells and endothelial cells underwent a fusion event or an intercellular transport event (Lu and Kang 2009; Pasquier et al. 2013). Cell fusion may contribute to the origin of cancer stem cells and acquisition of drug resistance. Specifically, glioblastoma-endothelial cell hybrids have been proposed to play a pivotal role in the perivascular microenvironment of glioblastoma and the maintenance of glioblastoma cancer stem cells (El Hallani et al. 2014). Further identification of the molecular signaling underlying the intercellular transport and elucidation of glioblastoma-endothelial hybrid function in glioblastoma progression can further our understanding of the angiogenesis of glioblastoma. However, it has been reported that brain microvascular endothelial cells show a distinct phenotype compared to endothelial cells isolated from umbilical cord, suggesting that closer physiological models may require the use of tissue-specific endothelial cells (Ye et al. 2014; Reinitz et al. 2015).

### Quick-fit shear flow and electric field co-stimulation chip for high throughput experiments

**Fig. 8:**
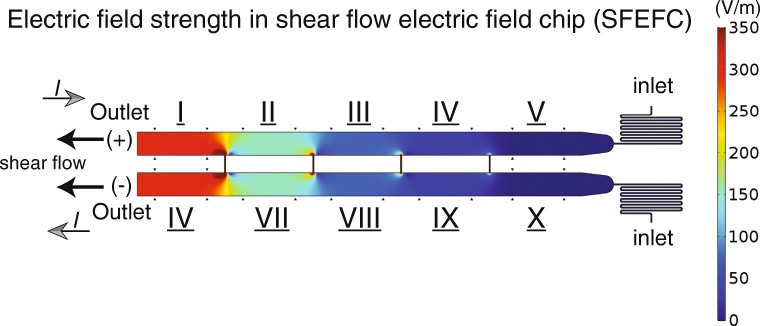
Numerical results of electric field strength in the shear flow and electric field co-stimulation microfluidic chip (SFEFC). SFEFC features 10 segments with coexistent electric field and shear flow. *I* denotes electric current. From sections I to IV, the shear flow flows against the electric current direction, while in sections VI to IX, the shear flow flows along the electric current vector

In the shear flow and electric field co-stimulation chip (SFEFC), 10 segments with combinations of shear flow (fixed shear stress of 1 Pa) and varying electric field strengths have provided possibilities for high-throughput experiments (see Fig. 8). Some key advantages of quick-fit SFEFC include: operator-friendly workflow, low dead volume, low reagent usage, air bubble free operation, air-tight sealing to sustain high flow rates, and reusable top PMMA interface chip that can be assembled with different PMMA/PDMS microchannel chips.

**Fig. 9:**
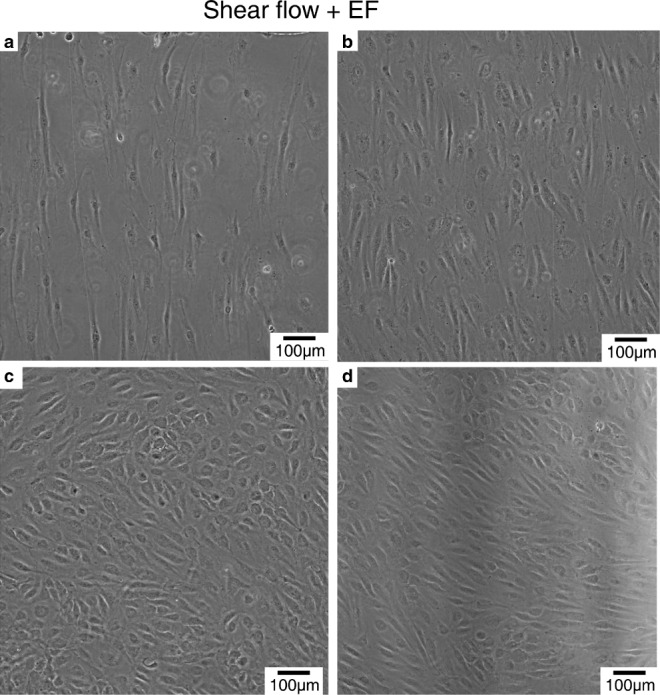
Microscopy images of endothelial cells cultured in sections I, II, III, and V in SFEFC. **a** Endothelial cells conditioned with shear flow (1 Pa) and 300 V m^− 1^. **b** Endothelial cells conditioned with shear flow (1 Pa) and 153.6 V m^− 1^ showing perpendicular alignment as a result of electrical stimulation. **c** Endothelial cells conditioned with shear flow (1 Pa) and 75.5 V m^− 1^. **d** Endothelial cells conditioned with shear flow (1 Pa) demonstrated more parallel alignment phenotypes

Although the sealing of SFEFC is reversible, it can withstand high flow rates necessary in microfluidic flow cells to create shear stress conditions similar to that in physiological hemodynamics (0.1–0.6 Pa in normal vein and 1.0–7.0 Pa in normal artery as reported by Malek et al. 1999). HUVECs cultured in SFEFC under simultaneous shear flow and electric field conditions demonstrated characteristic morphological changes (Fig. 9). The shear flow imposes a shear stress on adhered endothelial cells and induces an aligned morphology and a quiescent anti-inflammatory and anti-thrombotic phenotype (Uzarski et al. 2013). Electric field stimulation on endothelial cells instead induces a perpendicular aligned morphology with upregulation of a pro-angiogenic response and the release of VEGF (Zhao et al. 2004; Bai et al. 2011).

Under concurrent 5.4 mL h^− 1^ shear flow with 1 Pa shear stress and 300 V m^− 1^ dcEF stimulation, endothelial cells aligned perpendicular to the electrical current vector and the dcEF caused some cell death that rendered the endothelial cell layer patchy as shown in Fig. 9a. Strong perpendicular cell alignment was also evident in cells stimulated with concurrent 5.4 mL h^− 1^ shear flow and 153.6 V m^− 1^ dcEF (Fig. 9b). Under the fixed shear flow rate (1 Pa), perpendicular cell alignment by electrical field stimulation was not observed in cells conditioned with 75.5 V m^− 1^ electric field (Fig. 9c), suggesting that a minimum electric field stimulation on cells is required to induce the perpendicular cell alignment. Under the 5.4 mL h^− 1^ shear flow stimulation alone, the 1 Pa shear stress induced classical parallel alignment of HUVECs (Fig. 9d), consistent with prior observations (Buchanan et al. 2014; Abaci et al. 2014). Without any shear flow, the HUVECs exhibited no alignment preference (Fig. 4a). These results imply that the SFEFC in our experimental setup can support long-term shear flow and electrical stimulation conditioning of endothelial cells on chip (Fig. 1).

The alignment of HUVECs in SFEFC under simultaneous shear flow and electric field was further characterized by immunofluorescence staining against F-actin and CD31 (supplementary figure S.5). CD31 was expressed on conditioned endothelial cells. Under shear flow conditioning, endothelial cells displayed parallel alignment that was also demonstrated by the parallel orientation of actin stress fibers. When a co-existing electric field increased in strength, electrical stimulation induced cells to undertake a more perpendicular phenotype as the stress fibers became more perpendicularly oriented.

### Glioblastoma adhesion to endothelial cell layer conditioned by shear flow and electric field

**Fig. 10:**
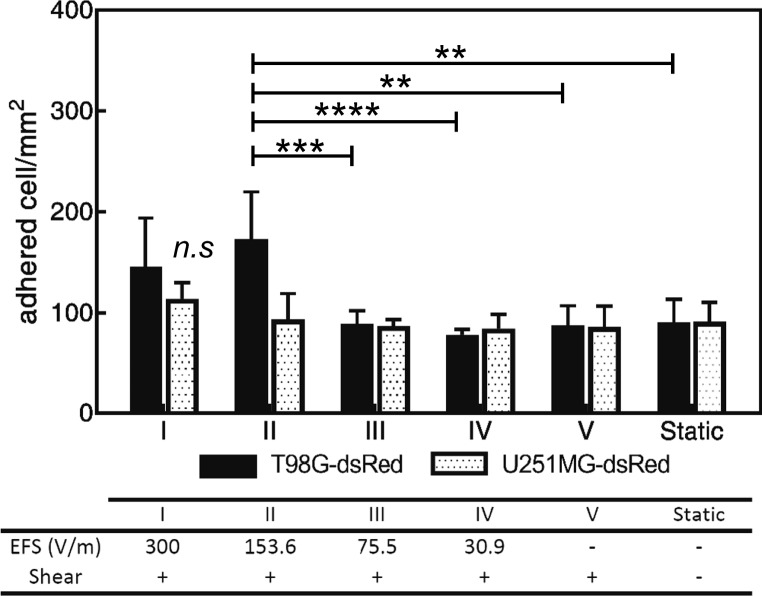
Adherence of glioblastoma cells to endothelium conditioned under shear flow and electric field or endothelium cultured in static flow on the SFEFC. ** denotes P < 0.01; *** denotes P < 0.001; **** denotes P < 0.0001

We next examined how simultaneous shear flow and electric field stimulation affected the adhesion of the two glioblastoma cell lines on the endothelial cells (Fig. 10). Only T98G-dsRed cells (not U251MG-dsRed cells, P > 0.05) showed increased adhesion to the endothelial cells preconditioned under 153.6 V m^− 1^ dcEF and 1 Pa shear stress when compared to two pre-conditioned control studies: shear-flow conditioned control (P < 0.01, section V of Fig. 10) and the static control (P < 0.01). As described in Section 3.2, the endothelial cells preconditioned under 300 V m^− 1^ dcEF and shear flow with 1 Pa shear stress were patchy due to some cell death at this electric field strength (Fig. 9a), which may contribute to the lower adhesion of glioblastoma cells (P > 0.05). Even though the endothelial cells conditioned in shear flow of 1 Pa and electric fields of 75.5 V m^− 1^ and 30.9 V m^− 1^ were confluent, the adhesion of both glioblastoma cells lines on pre-conditioned endothelial cells were similar when compared to the control groups. This suggests a dose dependent response of electric field conditioning which was observed in the adhesion of osteosarcoma to polyethylene substrate by Naegele et al. (1991) and migration of fibroblasts by Song et al. (2013). Future identification of the molecular targets that contribute to the difference of adhesion could facilitate molecular typing of glioblastoma cells and further our understanding on the cell-cell interaction between glioblastoma and the endothelial cells that may contribute to the metastasis (Wang et al. 2010; Lombard et al. 2015).

In addition, under the static condition, the adhesion of U251MG-dsRed was higher than that of the adhesion of T98G-dsRed cells (Fig. 6), but such difference was not observed in the on-chip static control (Fig. 10). The shear force from the gravity flow in on-chip glioblastoma adhesion experiments could contribute to this discrepancy. It is known that hydrodynamic flow could alter the adhesion dynamics and cell-cell contact. Quantitative study of glioblastoma-endothelial adhesion dynamics under a hydrodynamic flow can elucidate the underlying biophysical mechanism in future studies (Korn and Schwarz 2006).

In this work, applied electrical current induces perpendicular cell alignment and shear flow instigates a parallel alignment on endothelial cells, thus cells are subjected to competing physical cues. In the future, a microfluidic design with orthogonal electric field and shear flow configuration can be useful for investigating the synergistic cell alignment effect in a complex microenvironment.

## Conclusion

We demonstrated a quick-fit hybrid BMMD made of PMMA and PDMS that provided advantages over single-material counterparts, such as bubble prevention, user-friendliness, low dead volume, and air-tight sealing. The quick-fit design allows high experimental-throughput setup so that by reusing the top interface chip, multiple experiments can be performed sequentially by assembling and disassembling the quick-fit chip. The reagent waste was significantly reduced.

We verified the operation of quick-fit BMMD by conditioning endothelial cells on-chip to concurrent shear flow and electric field. No leakage or bubbles at high volumetric flow rate were observed. T98G-dsRed and U251MG-dsRed glioblastoma cell adhesion under static culture and shear flow with electric field-conditioned endothelium was examined. Angiopoietin 1 activation increased the adhesion of both glioblastoma cell lines on a statically cultured endothelial cell layer. T98G-dsRed glioblastoma cells also showed increased adhesion to an endothelial cell layer conditioned with intermediate electric field and shear flow. On the other hand, U251MG-dsRed showed no adhesion difference. Further identification and typing of adhesion molecules is expected to further our understanding of how glioblastoma interacts with endothelial cells. We envision that the quick-fit hybrid microdevice can be applied to study other cell-tissue interactions in controlled shear flow and electric field conditions. The quick-fit hybrid device can also aid in drug screening on cells conditioned under biomimetic conditions.

## Electronic supplementary material

Below is the link to the electronic supplementary material.

(PDF 14.0 MB)

(AVI 20.9 MB)
