# Supplementary material for: Glioblastoma adhesion in a quick-fit hybrid microdevice
Source: Biomed Microdevices. 2019 Mar 21;21(2):30. doi: 10.1007/s10544-019-0382-0 (PMC6428793; doi:10.1007/s10544-019-0382-0)
Supplement: Supplementary file 1 — (PDF 14.0 MB) [file 10544_2019_382_MOESM1_ESM.pdf]

Glioblastoma adhesion in a quick-fit hybrid microdevice

Hsieh-Fu Tsai[1,2], Kazumi Toda-Peters[1], Amy Q. Shen[1*]

[1] Micro/Bio/Nanofluidics Unit, Okinawa Institute of Science and Technology Graduate University, Okinawa, Japan

[2] Research Fellow of Japan Society for the Promotion of Science

∗ To whom correspondence should be addressed. Email: amy.shen@oist.jp

January 8, 2019

Supplementary Figures

List of Figures

S.1 A snapshot of the experimental setup of concurrent shear flow and electric field conditioning of endothelial cells in a "shear flow and electric field co-stimulation microfluidic chip (SFEFC)". . 2

S.2 The adherence of T98G-dsRed cells (red fluorescence) to endothelial cells (green fluorescence). Yellow fluorescence represents the intercellular transport between the two cells. 3

S.3 The adherence of U251MG-dsRed cells (red fluorescnece) to endothelial cells (green fluorescence). Yellow fluorescence represents the intercellular transport between the two cells. 4

S.4 The intercellular transport events observed in static condition. 5

S.5 Immunofluorescence staining of HUVECs conditioned with shear flow (shear stress fixed at 1 Pa) and electric fields at various strengths. 6

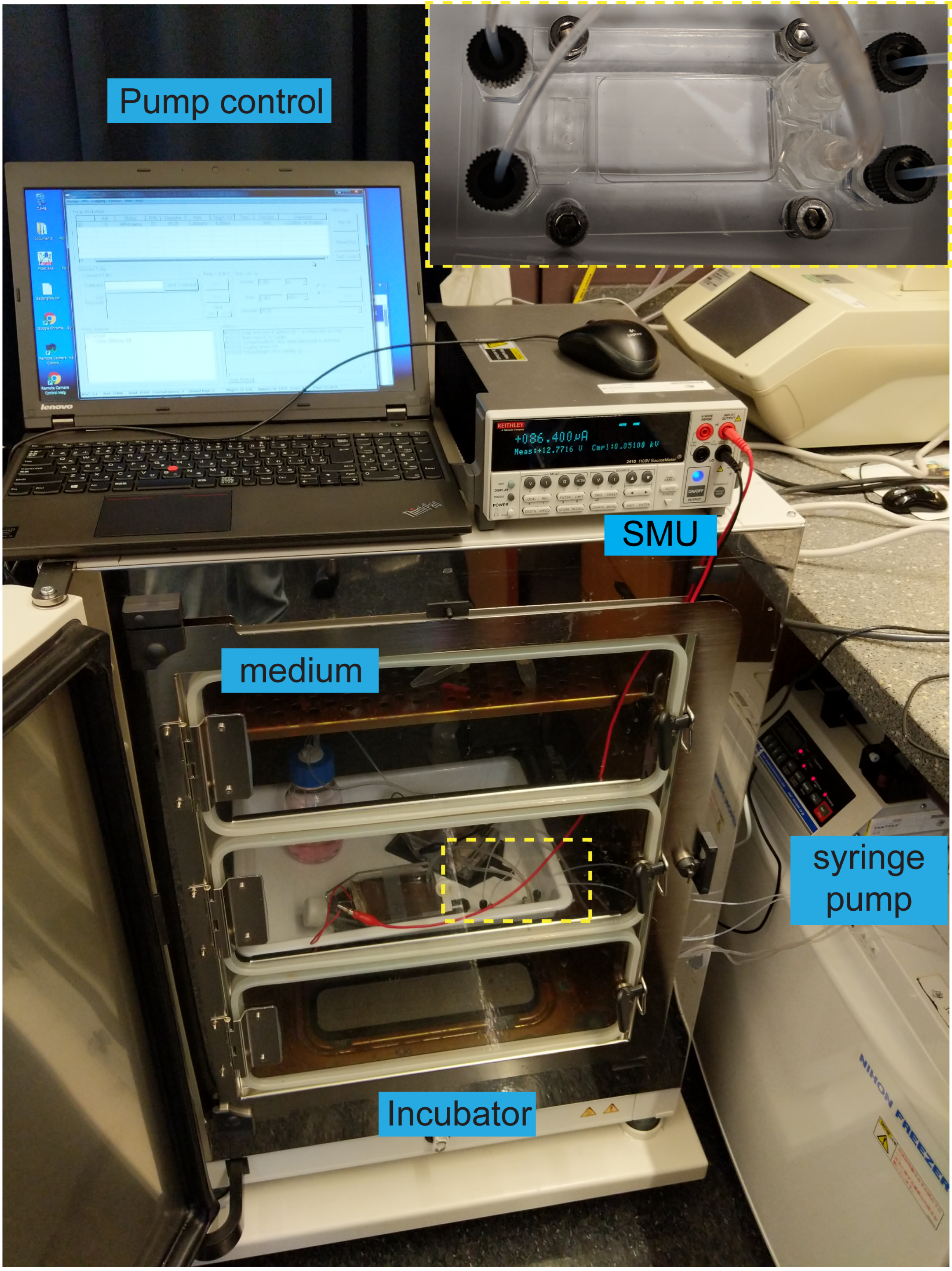

Fig. S.1: A snapshot of the experimental setup of concurrent shear flow and electric field conditioning of endothelial cells in a “shear flow and electric field co-stimulation microfluidic chip (SFEFC)”. Both the media bottle and the SFEFC chip are placed in a humidity controlled incubator for cell culture. The shear flow is imposed by withdrawing the media using a syringe pump with programmable control software. The electric field is applied by a source measure unit (SMU). The top right image in the yellow dash-box is the zoomed-in picture of the assembled quick-fit SFEFC.

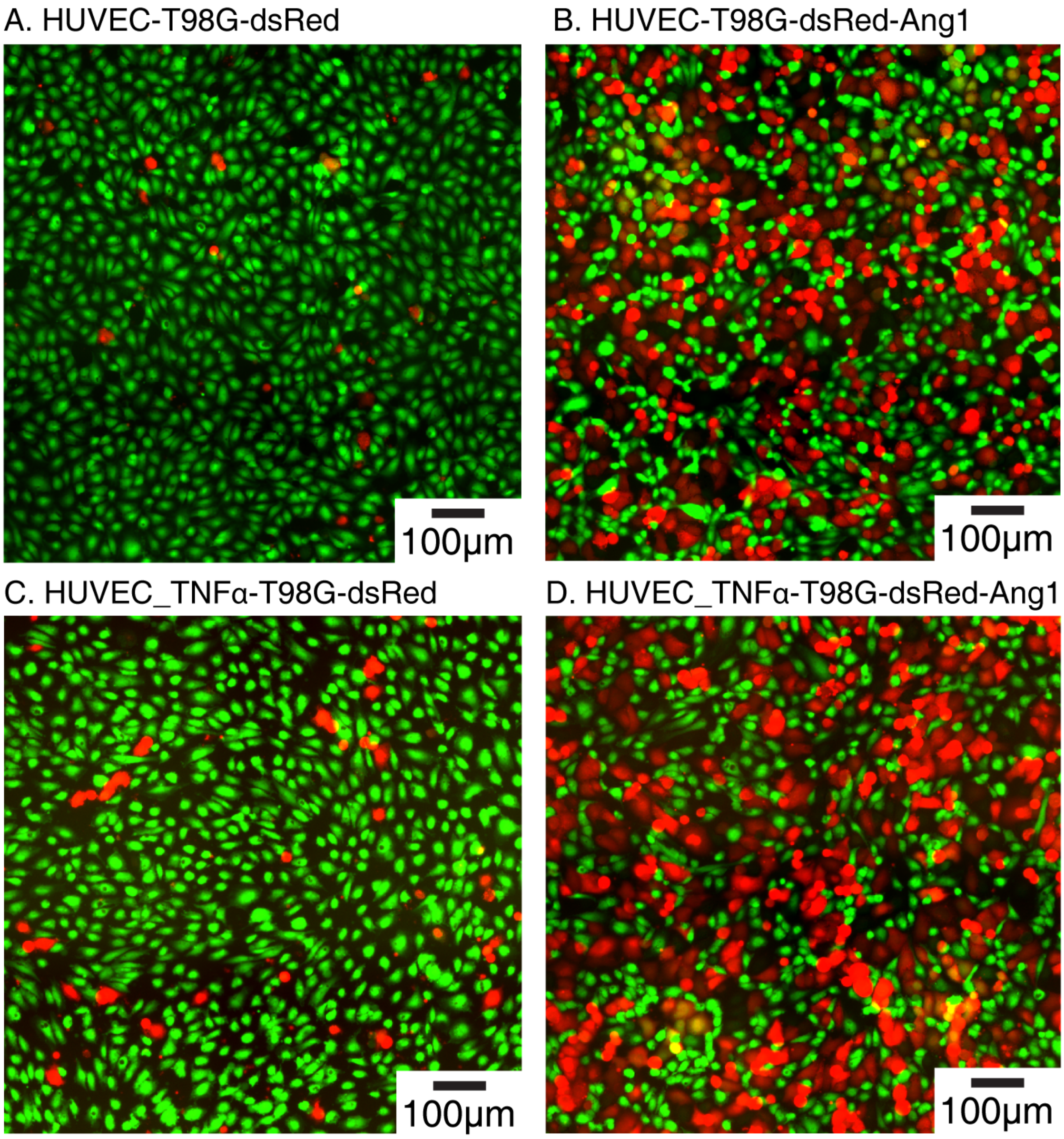

Fig. S.2: The adherence of T98G-dsRed cells (red fluorescence) to endothelial cells (green fluorescence). Yellow fluorescence represents the intercellular transport between the two cells. (A) T98G-dsRed adherence to HUVECs; (B) Increased adherence of Ang1-stimulated T98G-dsRed cells to HUVEC as evident by increased number of red fluorescent cells; (C) T98G-dsred adherence to TNFα-activated HUVECs; (D) Ang1-stimulated T98G-dsRed cells adherence to TNFα-activated HUVECs. Increased adherence are detected when the number of glioblastoma cells increases.

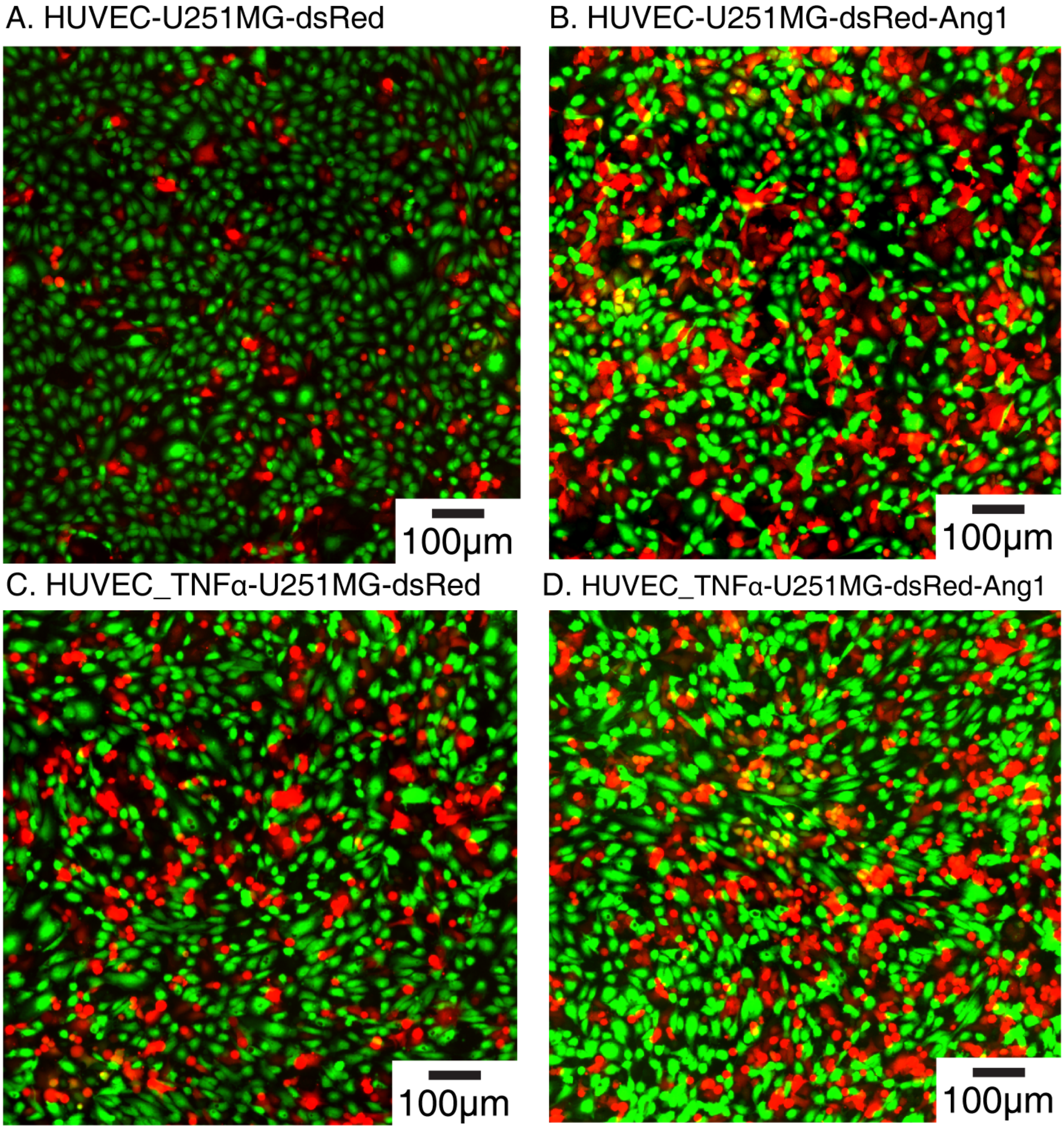

Fig. S.3: The adherence of U251MG-dsRed cells (red fluorescnece) to endothelial cells (green fluorescence). Yellow fluorescence represents the intercellular transport between the two cells. (A) U251MG-dsRed adherence to HUVECs; (B) Increased adherence of Ang1-stimulated U251MG-dsRed cells to HUVEC, as evident by increased number of red fluorescent cells; (C) U251MG-dsred adherence to TNFα-activated HUVECs; (D) Ang1-stimulated U251MG-dsRed cells adherence to TNFα-activated HUVECs. Increased adherence are detected when the number of glioblastoma cells increases.

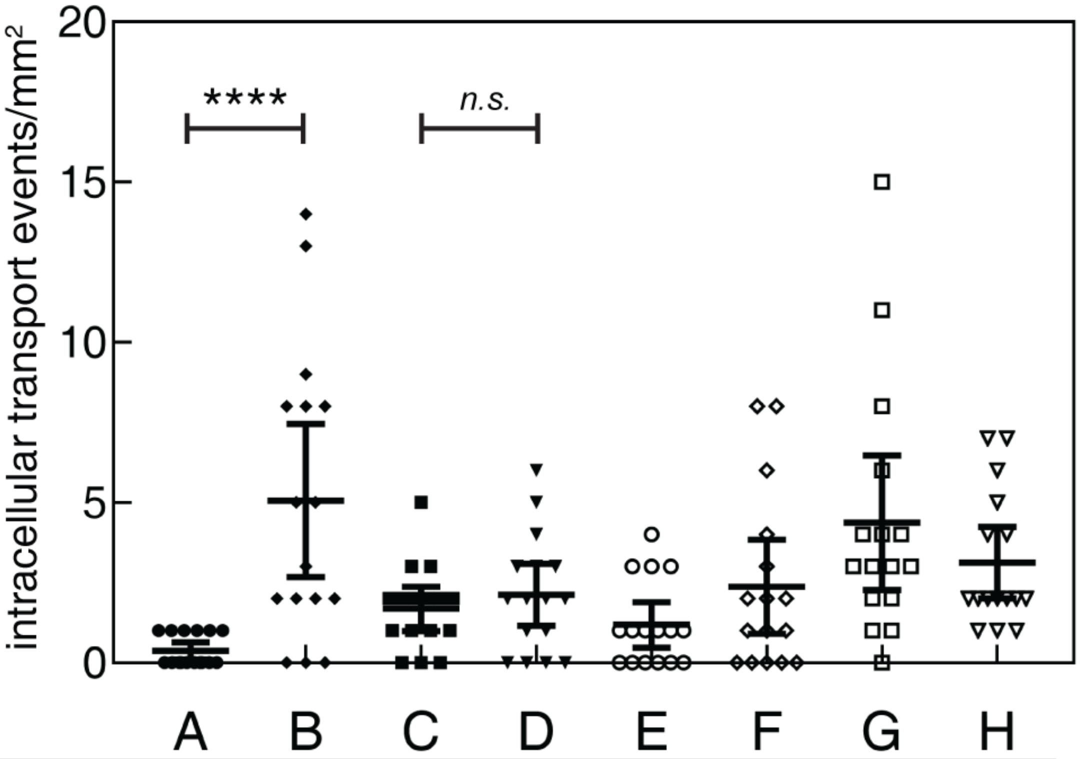

	A	B	C	D	E	F	G	H
Glioblastoma	T98G	T98G	U251MG	U251MG	T98G	T98G	U251MG	U251MG
Ang1	-	+	-	+	-	+	-	+
HUVEC	O	O	O	O				
HUVEC+TNFα					O	O	O	O

Fig. S.4: The intercellular transport events observed in static condition. **** denotes P<0.0001; n.s. denotes no significance. Signs of + and - denote whether the glioblastoma cells are stimulated with angiopoietin. Circle symbol denotes if the glioblastoma adhere to HUVECs or TNFα-stimulated HUVECs. The intercellular transport events in T98G cells increase when T98G are stimulated with Ang1 but no increase is observed in U251MG cells. TNFα stimulation has no statistically significant effect on intercellular transport events.

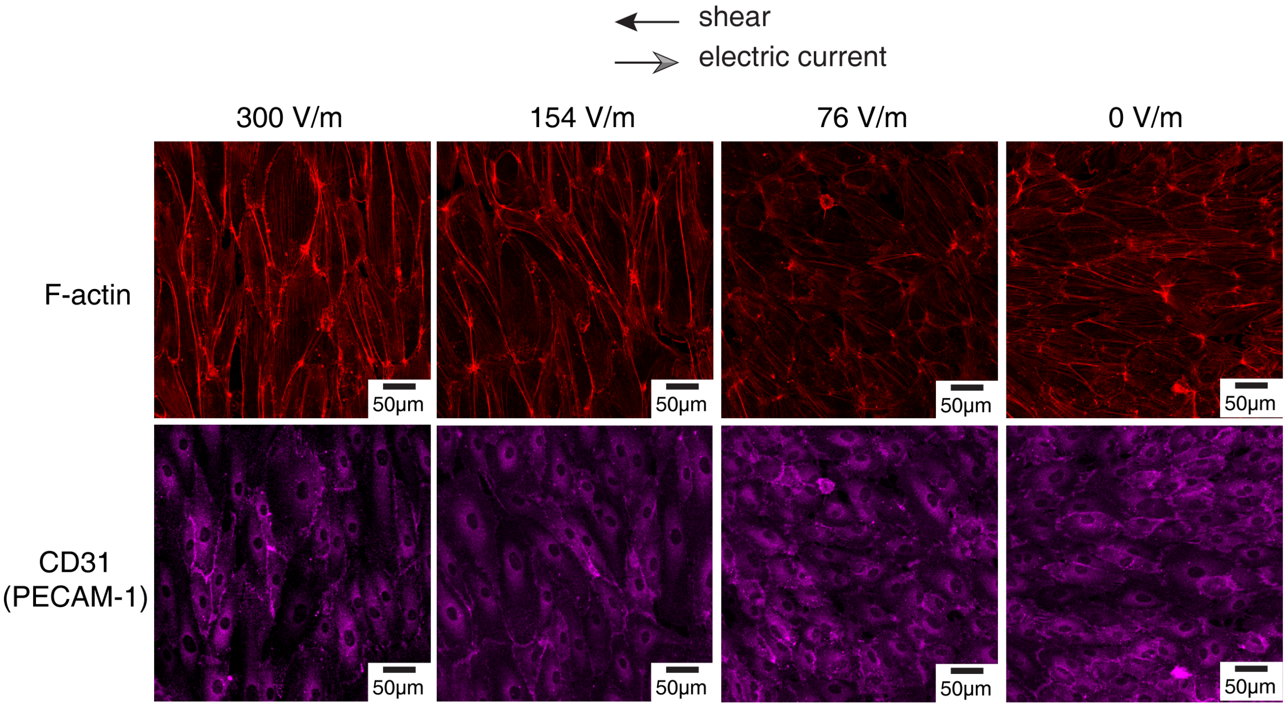

Fig. S.5: Immunofluorescence staining of HUVECs conditioned with shear flow (shear stress fixed at 1 Pa) and electric fields at various strengths. F-actin cytoskeleton staining by phalloidin shows stress fiber alignment under increasing electric field. CD31 is the platelet endothelial adhesion molecule expressed on endothelial cells. Evident perpendicular alignment of cells and the intracellular stress fibers are observed in cells stimulated with $154\,\mathrm{V\,m^{-1}}$ and $300\,\mathrm{V\,m^{-1}}$ electric field. The shear flow alone induces parallel alignment of cells and the stress fiber.

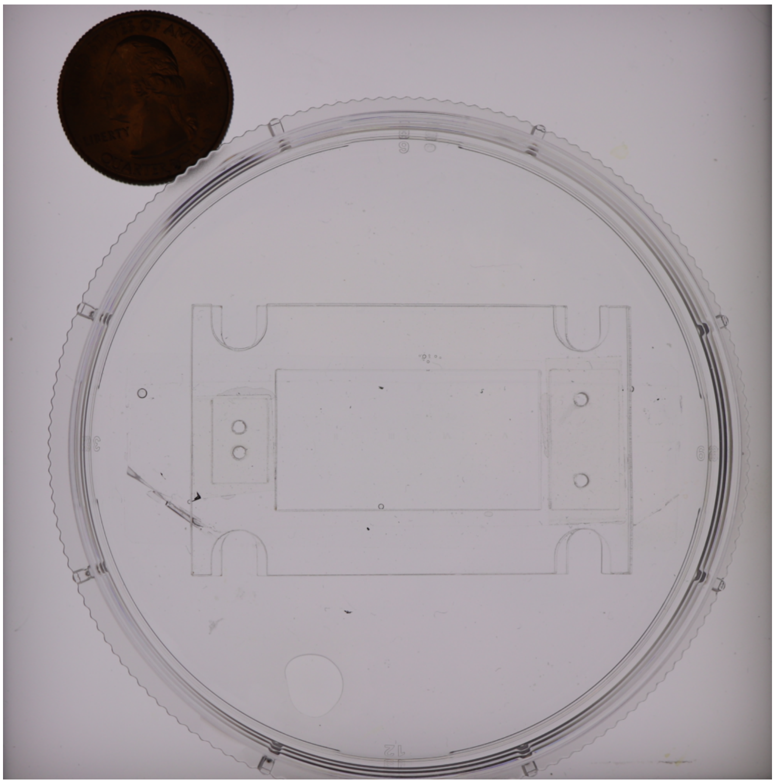

Video S.1: Video clip showing media replacement through gravity-driven flow by using micropipet tips. Different food dyes are dissolved in water to visualize the media in the SFEFC chip. A US quarter is used as scale.
